# Is there an interest in repeating the vaginal administration of dinoprostone (Propess®), to promote induction of labor of pregnant women at term? (RE-DINO): study protocol for a randomized controlled trial

**DOI:** 10.1186/s13063-019-3985-0

**Published:** 2020-01-08

**Authors:** P. Coste Mazeau, M. Hessas, R. Martin, J.-L. Eyraud, F. Margueritte, Y. Aubard, C. Sallee, F. Sire, T. Gauthier

**Affiliations:** 1 Department of Gynaecology and Obstetrics, Mother and Children’s Hospital, Limoges Regional University Hospitals, 8 Avenue Dominique Larrey, 87000 Limoges, France; 2 Clinical Investigation Center, CHRU Limoges, 2 Avenue Dominique Larrey, 87000 Limoges, France

**Keywords:** Induction of labor, Pregnancy, Dinoprostone, Oxytocin

## Abstract

**Background:**

Labor is induced in over 20% of women in France. Prostaglandins, especially intravaginal dinoprostone (Propess®), are widely used to initiate cervical ripening. If labor does not start within 24 h, there is uncertainty about whether to administer a second dinoprostone pessary or to use oxytocin to induce labor in order to achieve a vaginal delivery.

**Methods:**

RE-DINO is a prospective, open-label, multicenter, randomized superiority trial with two parallel arms running in six French hospitals. A total of 360 patients ≥ 18 years of age at > 37 weeks of gestation who exhibit unfavorable cervical conditions (Bishop score < 6) 24 h after placement of the first Propess®, with fetuses in cephalic presentation, will be included. Patients with premature membrane rupture, uterine scars, or multiple pregnancies will be excluded. Our principal objective is to determine whether placement of a second Propess® (followed by oxytocin [Syntocinon®], if necessary) in women for whom the first Propess® failed to induce cervical ripening increases the vaginal delivery rate compared to direct oxytocin injection. The vaginal delivery rate is therefore the primary outcome. The secondary outcomes are the induction failure rates and maternofetal morbidity and mortality.

**Discussion:**

This study may help in determining the optimal way to induce labor after failure of a first Propess®, an unresolved problem to date. This trial explores the effectiveness and safety of placing a second Propess® and may contribute to development of an obstetric consensus.

**Trial registration:**

Registered on 2 September 2016 at clinicaltrials.gov (identification number NCT02888041).

## Background

Perinatal surveys indicate that labor is induced in more than 20% of French women [1, 2]. Prostaglandins (PGs) [usually intravaginal dinoprostone (Propess®)] are widely used to initiate cervical ripening [3, 4].

The labor induction rate following placement of a single Propess® is 65–73%, with more than 80% of subsequent deliveries being vaginal [5, 6]. Currently, patients who have not entered labor 24 h after placing a Propess® are either induced by oxytocin (Syntocinon®) [7–9] or undergo cesarean section. Alternatively, a second Propess® is placed in several maternity hospitals [10] to reduce the rate of cesarean section (20.4% in France) [11]. A non-comparative retrospective monocentric study in 111 patients showed that placement of a second Propess® was associated with a vaginal delivery rate of 53.1% and no additional maternofetal morbidity or mortality [12]. Antonazzo et al. [13] performed a randomized trial with low patient numbers; the vaginal delivery rate was higher when a second Propess® was placed than when labor was induced by oxytocin. However, in the absence of a marketing authorization for use of a second Propess®, a prospective assessment is required.

Labor induction features medical stimulation of uterine contractions, effacing, and dilating the cervix to facilitate natural delivery [14]. In France, the labor of 22.7% of women is artificially induced [15, 16], and the indications may be maternal, fetal, or obstetric. Labor induction rates are rising all around the world and are likely to continue increasing since the publication in 2018 of the randomized trial of Grobman et al. that found that there is a decrease in the risk of cesarean with labor induction at 39 weeks of gestation (WG) rather than expectant management [17].

Cervical ripening refers to cervical preparation prior to artificial labor induction [1]. Many studies use “artificial induction” to describe cervical ripening [18]. We will use both terms interchangeably, as cervical ripening is an integral part of artificial labor induction.

The induction process is evaluated using the Bishop score [19], which considers the length, dilatation, position, and consistency of the cervix and the height of the mobile fetus. The score predicts the success of term labor induction [20]. A score < 6 is considered poor. Some teams define a score ≤ 3 as very poor, 4–5 as rather poor, and ≥ 6 as good [21]. Some professionals advocate the use of objective cervical ultrasound [22, 23], but this practice remains controversial [24]. Other predictors of the success of induction include multiparity, higher gestational age (> 41 + 4 WG) [25], maternal age (< 35 years) [26], and body mass index (< 30 kg/m^2^) [27, 28].

The recommendations regarding cervical ripening differ in terms of the chemicals used, route of administration, dosage, and monitoring modality. Mechanical induction is employed in 50% of maternity hospitals [18]. The double balloon (Cook®) is a single-use device placed for up to 12 h. The Foley® catheter is a single balloon catheter (n°18), and the Dilapan® is a cervical dilator.

Hormones are commonly used to induce labor. Oxytocin, in combination with amniotomy, is employed if the cervix is considered favorable (Bishop score ≥ 6), reducing the interval between induction and delivery [29]. If the cervix is unfavorable, oxytocin is also used to trigger labor but is not the first choice [29]. Natural (Propess®, Prostine®, and Prepidil®) and synthetic (Misoone®, Gymiso®, Nalador®, and Cervagem®) PGs have been used since 1968 to induce cervical ripening if the Bishop score is < 6.

PGs are widely used in France [30]. The Propess®, a vaginal patch featuring progressive continuous diffusion of 10 mg dinoprostone (0.33 mg/h), is placed for 24 h. Propess® use has increased greatly over time, from 10.2% [30] of maternity hospitals in 2001 to 89.1% today [10].

Dinoprostone in various forms (Propess®, Prostine®, and Prepidil®) exhibits a very good risk-to-benefit ratio [7]. According to the Higher Health Authority, intravaginal forms should be favored if PGs are used to induce labor [1]. Furthermore, the Propess® can obviously be removed at any time if uterine hypertonia (which may trigger fetal repercussions) develops, unlike prostaglandin gels (Prostine® and Prepidil®), which cannot be removed.

The National Institute for Health and Clinical Excellence states that labor induction using PGs increases 24-h vaginal delivery rates and reduces the need for cesarean section and epidural analgesia. Also, the maternal satisfaction rate is higher than when oxytocin (alone or with amniotomy) is used to induce labor [8].

Of all French maternity hospitals, 75.6% deliver oxytocin infusions regardless of the cervical condition (favorable or not) despite the risk of a cesarean section in instances of failure [10]. One study found that 65.4% of nulliparous patients with a Bishop score ≤ 3 underwent cesarean section after induction with oxytocin and amniotomy; in 66% of these cases, induction failed [31]. Moreover, a nulliparous status combined with a very unfavorable Bishop score imparted a 50% risk of induction failure, which decreased to 10% if the Bishop score was 4–6. One study involving 995 patients compared the use of single versus double doses of a vaginal prostaglandin E2 (PGE2) gel (2 mg PG); neither the cesarean nor instrumental extraction rate differed. Application of two PGE2 doses in multiparous women reduced the need for amniotomy and oxytocin [32].

Antonazzo et al. (2016) published the only randomized trial (albeit low patient numbers) demonstrating that the vaginal delivery rate increased significantly after a second vaginal application of dinoprostone (26/47, 55.3%) compared with oxytocin (16/47, 34.0%, *p* < 0.05), without any increase in neonatal morbidity [13].

In our Obstetrics and Gynecology Department (Limoges University Hospital), a second Propess® is sometimes applied in the absence of labor if the cervix remains unfavorable. In 2014, 44 of 65 patients (67.7%) who received a second Propess® delivered vaginally.

The French Higher Health Authority has issued dosage recommendations for PGE2 tablets and gels but does not mention the intravaginal diffusion systems, despite its widespread use (in one or two Propess®).

## Methods

### Objective and primary outcome

Our principal objective is to determine if the first Propess® does not induce cervical ripening and whether a second Propess® (followed by oxytocin (Syntocinon®)) if necessary) increases the vaginal delivery rate compared with that after direct oxytocin (Syntocinon®) injection.

The vaginal delivery rate is thus the primary outcome.

### Objectives and secondary outcomes

The secondary objectives and outcomes of our study are as follows:

- Comparison of induction failure (the absence of labor 24 h after placement of the second Propess® [cervical dilatation still < 3 cm]) between the test and control groups (cervical dilatation < 3 cm) despite 6 h of Syntocinon® infusion (one ampoule), amniotomy, and the presence of regular uterine contractions.
- Comparison of maternal morbidity and mortality between the test and control groups. We will record:
  - labor duration (time in minutes from attainment of 3 cm cervical dilatation to delivery),
  - indications for cesarean section,
  - all instances of instrumental extraction,
  - maternal hospitalization duration,
  - peri-delivery hemorrhage (> 500 ml) and its treatment,
  - complete uterine rupture,
  - transfer to intensive care units,
  - death.
- Comparison of fetal morbidity and mortality between the test and control groups. We will record:
  - Apgar scores < 7 at 3, 5 min and < 9 at 10 min,
  - fetal acidosis (umbilical arterial pH < 7.15 and < 7, lactate levels > 5, base excesses > 12),
  - all instances of amniotic fluid containing meconium at birth,
  - transfer to the neonatal intensive care unit,
  - fetal/neonatal death.

### Inclusion criteria

The inclusion criteria are:

Patients ≥18 years who have already had a first Propess®, within 24 to 36 h (before signing the consent) because of medical indications, a term pregnancy (> 37 WG), unfavorable cervical condition (Bishop score < 6), and intact membranes 1 h before inclusion. The fetus must be in cephalic presentation and the patient affiliated with a French social security scheme. Patients must sign the consent form.

### Exclusion criteria

The exclusion criteria are:

- multiple pregnancy,
- prior uterine scar (previous cesarean deliveries or myomectomy)
- any contraindications to the epidural anesthesia,
- contraindications to Propess® (a recent history of pelvic inflammatory disease or PG hypersensitivity)
- abnormal uterine contractility or an abnormal fetal heart rate triggering the removal of the first Propess®, or any adverse effect while the first Propess® is in place (anaphylactic shock or disseminated intravascular coagulation).
- contraindications to Syntocinon® (oxytocin hypersensitivity, any cardiovascular disorder, or severe toxemia of pregnancy)
- induction of premature membrane rupture,
- fetus with intrauterine growth retardation (< 3rd percentile) or macrosomic presentation (> 97th percentile),
- severe abnormal fetal heart rate
- induction due to fetal death in utero, medical termination of the pregnancy, or fetus presenting a lethal fetal pathology
- patients under guardianship or curatorship or in custody.

### Trial design

This is a multicenter, randomized, open-label, superiority trial with two parallel arms.

If labor has not commenced 24 h after placement of the first Propess®, patients will be asked to give written informed consent and then will be electronically randomized by a study investigator (doctor) to the control (oxytocin and peridural analgesia if the patient wishes) or experimental (a second 24-h Propess® prior to oxytocin if necessary and peridural analgesia if the patient wishes) group. Written informed consent will be obtained from all participants by a study investigator (doctor) before randomization.

Randomization (ratio 1:1) will be carried out centrally via the Center for Epidemiology, Biostatistics and Research Methodology (CEBIMER) computer platform (Ennov Clinical®) accessible 24 h a day, 7 days a week. To randomize a patient, the investigator must connect to the platform (secure connection) and after a phase of verification of the eligibility criteria, the randomization will be performed automatically.

The allocation will be performed by minimization, which provides similar distribution of selected factors between control and experimental groups. Randomization lists are not set up in advance and the first participant is randomly allocated. For each following participant, the treatment allocation that minimizes the mismatch on the five selected factors between groups will be identified.

It will be a randomization by minimization on the following factors:

- Bishop’s score (< 3, 3–5),
- body mass index (< 30, ≥30 kg/m2)
- parity (nulliparous, multiparous)
- age (< 40, ≥ 40 years old)
- center

First, a draft randomization list will be established by the methodologist and validated by the data manager. Then, the final randomization list will be performed and will be kept securely by the CEBIMER.

The follow-up data will be collected up to 10 days from delivery. This is not a blind study because of the galenic differences and the different methods of administration of the two treatments. As the induction of labor is necessary and justified for medical reasons, it is not possible to administer placebo.

### Interventions

In the control group, 5 IU of oxytocin will be diluted in 49 ml of physiological saline and will be started at 2.5 mIU/min, a flow rate of 1.5 ml/h with an increase of 2.5 mIU every 20 to 30 min up to 12 ml/h. In case of abnormal fetal heart rate or abnormal uterine contractions, oxytocin will be stopped momentarily. In case of induction failure (cervical dilatation remaining < 3 cm despite 6 h of oxytocin = 1 syringe and the presence of regular uterine contractions and amniotomy), the patient must have a cesarean section. In total, oxytocin is administered to induce labor for 6 h and then if the labor begins, oxytocin can be continued for one more syringe in case there is a lack of uterine contractions or labor stagnation.

In the experimental group, the second Propess® must be removed after 24 h or earlier if labor begins, or in case of abnormal uterine contractions, abnormal fetal heart rate or poor material tolerance. If oxytocin is needed in the experimental group after 24 h of the 2nd Propess®, it should be delivered in the same way as in the control group. If the labor does not begin (cervical dilatation remaining < 3 cm despite second Propess® + 6 h of oxytocin = 1 syringe and the presence of regular uterine contractions and amniotomy), the patient must have a cesarean section. In case the second Propess® falls off, a 2-h-long wait is tolerated. If labor does not start and if the fall occurred before 18 h of use, another Propess® should be inserted for a duration which should not exceed 24 h of cumulative exposure. In case it falls after 18 h of use, induction of labor should continue through oxytocin.

Both treatments will be labeled and supplied to the different centers and stored according to the manufacturers’ recommendations: between − 10 and − 20 °C for Propess® and between + 2 and + 8 °C for oxytocin. A regular temperature verification must be done to ensure the good conservation of the products. Antalgic medications are authorized. Only Risordan® and Natispray® are allowed to correct abnormal uterine contractions. No other medical or mechanical labor-inducing means are permitted.

### Participant’s timeline

**Fig. 1:**
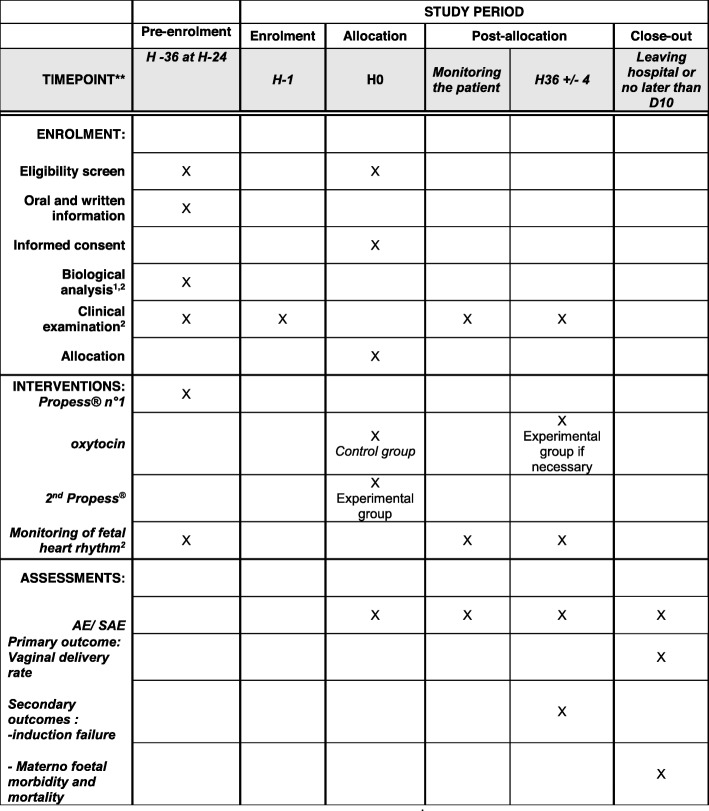
SPIRIT figure. Schedule Of Enrolment, Interventions, And Assessments. ** H = hours; D = days. AE = adverse events / SAE = serious adverse events. ^1^ Complete blood counts, blood coagulation and search of irregular agglutinin. ^2^ By the midwife

A brief Standard Protocol Items: A Schedule Of Enrolment, Interventions, And Assessments (in accordance with the Recommendations from Interventional Trials (SPIRIT)) is provided in Fig. 1.

### Sample size calculation

The number of participants is based on the primary outcome calculation for a comparative superiority test. Based on a local retrospective study (55% vaginal delivery rate after Propess® and 70% after two Propess® placements and Syntocinon®) performed prior to that of Antonazzo [13], the 15% between-group difference indicates that to afford an 80% power to detect a specific difference at the 5% significance level (two-sided test), 176 patients per group are required in a two-group scenario (nQuery Advisor 7). As the rate of non-evaluable patients (early drop-out, violation of inclusion or exclusion criteria, lack of compliance, intercurrent illness, comedication which was not allowed by the protocol) is expected to be very low in this study due to the nature of the primary outcome (vaginal delivery), we believe that a total of 360 (180 per group) will be an adequate sample size for the study.

### Data collection and management

Data on every outcome will be collected via an electronic case record form (ECRF) built by the Center for Epidemiology, Biostatistics and Research Methodology (CEBIMER) of the University Hospital of Limoges with the software CLINSIGHT (CLINSIGHT company). ERCFs will be completed by the investigator and a clinical trial technician. This database will be hosted on a server dedicated to CEBIMER CHU Limoges in secure premises. The promotor (University Hospital of Limoges) will have access to all data. A daily backup of the database will be performed. Periodic data completeness and quality checks will be performed by the study team by means of internal audits.

As it is anticipated that participants will only stay in the hospital for a maximum of 10 days after delivery, there are no strategies in place for collecting other outcome data. The reasons for not being eligible or not wanting to take part in the study will be recorded and reported in a CONSORT flow diagram, but the data for these participants will not be collected for analysis.

Patients withdrawing their consent after randomization will be noted in the CONSORT Flow-Chart and we will perform analyses intended to treat. In accordance with Law No. 2002–303 of March 4, 2002, patients will have access to the overall results of the research at their request.

### Serious adverse events

Serious adverse events (SAEs) will be any medical occurrence that results in death, is life-threatening, causes or prolongs hospital admission, results in persistent or significant disability or incapacity, or results in congenital anomaly. In the study protocol, maternal and fetal/neonatal SAEs will be divided into a group of “expected SAEs” explained by and related to the two treatments (Propess® and oxytocin) and “unexpected SAEs”. An immediate notification of all of these SAEs must be sent by the investigator to the local Clinical Trial Vigilance Unit and the promotor. Each year, the promotor sends to the “Agence Nationale pour la Sécurité du Médicament et des Produits de Santé” (ANSM) and to the Committee for the Protection of Person (CPP) a safety report of the SAEs.

### Monitoring of the study

A project leader of the Medical Affairs and Innovation Department will be responsible for the coordination and management of the trial (regulation, general coordination of the study, financial and administrative management, monitoring of inclusions). A clinical research associate monitor is sent regularly to each center by the promotor. There is a monitoring plan defining the frequency of the visits and the elements to monitor: consents, principal outcome, eligibility criteria, adverse events and SAEs, treatments.

The establishment of an independent monitoring committee was decided because the study involves pregnant women using a drug outside the marketing authorization. This committee, composed of three people outside of the Limoges University Hospital (one obstetrician gynecologist and two pharmacologists) met at the start of the study and will meet every 60 patients. The committee is there to ensure the absence of unbalanced treatment that may lead to an excess of morbidity and mortality in the groups. So, the role of this independent monitoring committee is to monitor the study data and safety of participants. The opinion of the committee is conveyed to the promotor (University Hospital of Limoges) and the project team.

Thus, any amendment to the protocol must be approved by the research team, the promotor, and the monitoring committee before being submitted to CPP and ANSM.

### Statistical analysis

Data will be analyzed by the UVEC (pharmacovigilance unit) for security and vigilance analyses and local CEBIMER for the main statistical analyses using SAS 9.3 (SAS Institute Cary, NC). *p* values < 0.05 will be considered statistically significant. Our analysis will be conducted using the intention-to-treat principle and reporting will follow CONSORT 2010 recommendations.

Descriptive statistics of continuous data will be reported as means ± standard deviations or medians and interquartile ranges depending on the distribution of the variable. Dichotomous and categorical data will be presented by numbers and percentages. The primary outcome will be reported with numbers and percentages in each group. The analysis of the primary outcome will be conducted by comparing the proportions of vaginal delivery between the two groups with the use of a Chi-square or Fisher’s exact test depending on the theoretical numbers. The effect seize will be estimated using odds ratio and its 95% confidence interval.

Secondary analyses will be computed in order to compare failure induction of labor, maternal and fetal morbimortality between the two groups of randomization by using a Chi-square or a Fisher’s exact test depending on the theoretical number for dichotomous or categorical outcomes and a Student’s *t* test or a non-parametric Mann–Whitney test for continuous outcomes depending on the distribution of the variable.

We will use the Shapiro–Wilk test for testing whether variables are normally distributed.

All data analysis will be performed unblinded according to a pre-established statistical analysis plan drafted prior to access to the data and reported in a document signed by all authors. This statistical analysis plan will be updated before data lock for the final analysis.

### Investigation sites

RE-DINO operates in six French centers, selected for their obstetrical practices (routine use of a second Propess®), wish to be involved, and employment of high-quality research and clinical staff. Each center performed an in-house feasibility study. At the beginning, we will include at least 12 patients per month, thus at least two per month per center, over 30 months in several hospitals/university hospitals: Limoges, Clermont–Ferrand, and Marseille University Hospitals and Brive, and Tulle Hospitals. Initially, Blois Hospital was an investigative center but due to the low number of inclusions the center was closed and replaced in the study by Chambéry Hospital.

Central ethical approval has been confirmed from the Committee for the Protection of Personne of Sud-Ouest Outre-Mer IV (ref approval no. CPP16–030-PP) and we started recruiting at other centers in the trial only after local ethical approval was obtained.

### Authorizations and dissemination

All investigators will strictly follow the study protocol prepared by Limoges University Hospital. All investigators have obtained their certificate of “Good Clinical Practice”. This research is funded by a 2015 PHRC-I grant (Interregional Clinical Research Hospital Protocol) allocated to the promotor (= sponsor) of the study: Limoges University Hospital. The promotor, located at 2 avenue Dominique Larrey 87,000 Limoges, assumes overall responsibility for the initiation and management of the trial. The principal investigator is Dr. P. Coste-Mazeau, gynecologist/obstetrician at Limoges Regional University Hospitals. The funder (French Ministry of Health) does not have any authority in study design; collection, management, analysis, and interpretation of data; writing of the report; and the decision to submit the report for publication. RE-DINO was approved by the Committee for the Protection of Personnel of Sud-Ouest Outre-Mer IV on 10 June 2016 (ref approval no. CPP16–030-PP) and authorized by the “Agence Nationale pour la Sécurité du Médicament et des Produits de Santé” (ANSM) on 21 June 2016. The study has been entered in the clinicaltrials.gov website (no. NCT02888041).

The dissemination of the results will also be through international publications and will be reported in national and international meetings.

## Discussion

We seek to answer a major clinical question. An increase in the number of vaginal deliveries in the experimental group (treated via placement of a second Propess®) may indicate how to decrease the rising nationwide cesarean rate. In addition, vaginal delivery reduces the length of maternal hospitalization (3 days versus 5–6 days after a cesarean section), thereby lowering the costs of care borne by society.

Currently, the use of only one Propess® is authorized. Although many teams place a second Propess® if the first has no effect, no consensus, recommendations, or comparative data are available. We will deliver objective evidence that may contribute to the establishment of a national protocol and unification of obstetric practices.

## Trial status

At the time of writing, the protocol is on its ninth version. Recruitment into the trial began on 30 December 2016. At the time of study design, the expected recruitment duration was 30 months. However, due to slower-than-anticipated accrual, the recruitment period has been extended to January 2022. On 10/22/2019, 110 patients have been randomized.

A populated SPIRIT checklist is provided in Additional file 1.

## Supplementary information

**Additional file 1.** SPIRIT 2013 Checklist: Recommended items to address in a clinical trial protocol and related documents.

## Data Availability

Not applicable.

## Abbreviations

ANSM: “Agence Nationale pour la Sécurité du Médicament et des Produits de Santé”
CEBIMER: Center for Epidemiology, Biostatistics and Research Methodology of the University Hospital of Limoges
CONSORT: Consolidated Standards of Reporting Trials
CPP: Committee for the Protection of Personnel
ECRF: Electronic case record form
PG: Prostaglandin
PGE2: Prostaglandin E2
PHRC-I: Interregional Clinical Research Hospital Protocol
SAES: Serious adverse events
SPIRIT: Recommendations for Interventional Trials
UVEC: Unit of vigilance of clinical trials (pharmacovigilance unit)
WG: Weeks of gestation
